# Research progress of glucocorticoid resistance in chronic rhinosinusitis with nasal polyps: A review

**DOI:** 10.1097/MD.0000000000036024

**Published:** 2023-11-17

**Authors:** Langlang Chen, Xin Fan, Lina Yang, Lu Han, Ningbo Wang, Ka Bian

**Affiliations:** a Department of Otolaryngology Head and Neck Surgery, Tangdu Hospital, Air Fourth Medical University, Xi’an, China; b Medicine College of Yan’an University, Yan’an, China; c State Key Laboratory of Oral & Maxillofacial Reconstruction and Regeneration, School of Stomatology, Air Fourth Medical University, Xi’an, China.

**Keywords:** glucocorticoids, nasal polyps, receptors, resistance, rhinosinusitis

## Abstract

Chronic rhinosinusitis with nasal polyps (CRSwNP) is one of the common chronic inflammatory diseases in otolaryngology. Glucocorticoid (GC) acts as the first-line drug for the treatment of CRSwNP in clinical practice, and they play an irreplaceable role in reducing nasal mucosal inflammation and restoring the normal physiological function of the nasal mucosa. However, many patients are still insensitive to GC treatment, known as GC resistance, which leads to poor control of the disease, and the underlying mechanisms are still not fully elucidated. This article provides a comprehensive overview of the research progress of GC resistance of patients with CRSwNP in recent years.

## 1. Introduction

Chronic rhinosinusitis (CRS) is an inflammatory disease of the nasal cavity and paranasal sinuses with a prevalence of 5% to 15% in the general population, which manifests as nasal obstruction, viscous or mucopurulent nasal discharge, and may be accompanied by facial pain and olfactory dysfunction for more than 12 weeks.^[1]^ Clinically, CRS is divided into CRS with nasal polyps (CRSwNP) and CRS without nasal polyps (CRSsNP) depending on the presence or absence of nasal polyps, which demonstrate distinct tissue remodeling patterns and inflammatory cytokine profiles.^[2,3]^ Glucocorticoids (GCs) are recommended as a mainstay treatment for CRSwNP due to their powerful anti-inflammatory function, which can effectively alleviate mucosal inflammation and edema, improve symptoms, and to a certain extent, shrink or eliminate polyps size and increase the volume of the nasal cavity.^[4–6]^ Although GC therapy is valid for most cases, the response of CRSwNP patients to GC varies widely due to disease heterogeneity, referred to as GC resistance (GCR).^[7,8]^ To date, the mechanisms underlying the resistance of GC in CRSwNP patients are not yet clear, and this article systematically reviews the relevant research on GCR CRSwNP, providing an active reference for the treatment and prognosis of GC-resistant patients with CRSwNP.

## 2. The mechanism of GC in the treatment of CRSwNP

GC is a type of steroid hormone mainly secreted by the zona fasciculate of the adrenal cortex, regulated by the hypothalamic-pituitary-adrenal axis. The glucocorticoid receptor (GR) mediates the anti-inflammatory effect of GC, which is primarily utilized as an anti-inflammatory drug for the treatment of various diseases. GCs enter the cytoplasm of cells via membrane diffusion, where they bind to GRs and exert biological effects by increasing the expression of anti-inflammatory genes or inhibiting the expression of inflammatory genes.^[9]^ GRs are bound to molecular chaperones such as heat shock protein 90 (HSP90) to maintain their normal spatial structure.^[10]^ The binding of GC to its receptor causes the formation of the GC-GR complex, resulting in a conformational change of the GR and exposure of the activated receptor’s Deoxyribonucleic Acid (DNA) binding domain. The GC-GR complex is then transported into the nucleus, where it regulates gene transcription by binding to the glucocorticoid response element (GRE) at specific DNA sites.^[11,12]^ This genomic mechanism is responsible for the anti-inflammatory effects of GCs.

Evidence suggests that CRSwNP is linked to greater infiltration of eosinophils in tissue and increased T helper 2 (Th2) cytokine expression,^[13,14]^ which contributes to its pathogenesis. Due to their anti-inflammatory and immunomodulatory effects, GCs are a mainstay treatment for nasal inflammatory diseases. Administration of GCs can be via oral or intranasal routes. Studies have shown that GCs play a crucial role in reducing nasal mucosal inflammation and restoring normal physiological function.^[15,16]^ GCs exert a therapeutic effect by reducing eosinophil infiltration^[17]^ and inducing eosinophil apoptosis^[4]^ in nasal polyp tissue, ultimately alleviating the inflammatory response caused by eosinophils. This represents the non-genomic mechanism by which GC treats nasal polyps. Furthermore, GCs also exert their therapeutic effects on CRSwNP by inhibiting inflammatory mediators and modulating T cells’ immune responses, particularly those involving Th2 cells and related cytokines.^[18]^ Studies have demonstrated that GCs can significantly decrease the levels of eosinophil cationic protein, monocyte chemoattractant protein 4, interleukin-5 (IL-5), IL-13, and Immunoglobulin E in nasal secretions of nasal polyp patients.^[19,20]^ In addition, GCs also can suppress the expression of numerous cytokines, such as IL-1β, IL-6, TNF-α, growth factors including GM-CSF, and TGF-β, as well as chemokines belonging to the CXC and CC families.^[21,22]^

## 3. Current status of GCR in patients with CRSwNP

In the 1970s, Mygind et al^[23]^ first reported the use of betamethasone dipropionate in treating CRSwNP, which demonstrated significant efficacy and marked the beginning of the GC era in CRSwNP treatment. The current management for CRSwNP is a combination of drug therapy and surgical intervention aimed at both managing inflammation in the nasal cavity and sinuses and addressing the underlying causes of inflammation. In clinical practice, GCs are often recommended as first-line treatment for CRSwNP, receiving an A-level recommendation in the European Position Paper on Rhinosinusitis and Nasal Polyps guidelines.^[1]^ Despite its widespread use, studies have shown that nasal and oral GCs may only be effective in 50% to 80% of patients with CRSwNP, with some patients exhibiting resistance to GC treatment.^[24,25]^ Patients who respond to GC treatment may still tend to relapse after discontinuing conservative therapy. Furthermore, the longer the discontinuation period, the higher the likelihood of relapse. If conservative treatment fails, surgical intervention may be necessary to address CRSwNP. Functional endoscopic sinus surgery (FESS) can help alleviate obstructions, improve sinus drainage, and reduce inflammation. However, it is important to note that surgical treatment is not a one-time cure for CRSwNP. While FESS can provide a cure or mitigate symptoms in some patients with nasal polyps, at least 20% will require a second surgery.^[26]^ Moreover, the longer the follow-up period, the greater the likelihood of relapse and the need for additional operations. A 12-year follow-up study found that 78.9% of CRSwNP patients experienced relapse, with 36.8% requiring a second surgery.^[27]^

The concept of GCR in lymphoma research was first proposed by McPartland et al^[28]^ in 1977. In 1993, Smith et al^[29]^ reported that despite systemic drug or surgical treatment, approximately 12% of patients still experienced persistent inflammation in their nasal cavities and sinuses. Recent years have seen an increase in studies on the role of GCR in asthma. Clinically, GC-resistant asthma is defined as asthma patients who have been administered sufficient doses and duration of GC treatment (such as prednisone 40 mg/d for 2 weeks), yet exhibit a predicted FEV1 of less than 75%, with FEV1 improvement not exceeding 15%.^[30]^ However, there is currently no standard definition for GC-resistant CRS. The Jankowski scoring system is used in some studies to assess GC-resistant CRS before and after continuous nasal GC treatment (budesonide 200 µg/d) for 1 month. If the score improvement for nasal symptoms, olfactory improvement, peak nasal inspiratory flow rate, and nasal polyp size does not exceed 1 point, the patient is diagnosed with GCR.^[31]^ Some scholars define CRSwNP patients with GCR as those whose clinical symptoms and nasal polyp scores are unable to reduce more than 1 nasal polyp endoscopic score after receiving standardized nasal glucocorticoid treatment for 3 months and continuing oral glucocorticoid treatment for 15 days (with dexamethasone dosages of 1 mg/kg/d for 8 days and 0.5 mg/kg/d for 7 days).^[32]^

Despite standardized drug and surgical treatments, recurrent nasal polyps remain a challenge. GCR significantly impacts patients’ quality of life and requires substantial medical resources.

## 4. Pathogenic factors contributing to GCR

The etiology of GC-resistant CRSwNP is still not clear, and multiple factors are involved in its pathogenesis, which can be divided into 3 aspects: local factors, systemic factors, and patient-related factors.

### 4.1. Local factors

#### 4.1.1. Bacterial biofilm.

Bacterial biofilm refers to a bacterial community formed by mutual adhesion through the production of extracellular polysaccharide matrix in an environment unfavorable for bacterial growth.^[33]^ Bacteria are continuously released from the biofilm. Studies have found that about 77% of patients who have undergone endoscopic sinus surgery have bacterial biofilms,^[34]^ which are a significant contributor to chronic and persistent infection of the nasal mucosa. It has been found that bacterial biofilms not only play a role as infectious pathogens, but also as antigens, superantigens, adjuvants, toxins, and inflammatory factors to promote the occurrence and development of CRS.^[35]^ Furthermore, bacteria in biofilms are 500 to 1000 times more resistant to antibiotics than free-floating bacteria, showing significant resistance to both antibiotic treatment and host immune defenses.^[36]^ In addition, patients with CRS accompanied by bacterial biofilms had more serious conditions before surgery and were prone to persistent mucosal inflammation after surgery, making it difficult for GC to achieve corresponding effects during use.^[37]^

#### 4.1.2. Superantigen.

Research has confirmed that patients with CRSwNP typically have infections within the nasal cavity that are caused by aerobic and facultative anaerobic bacteria. The most common pathogenic bacteria include staphylococci, Streptococcus pneumoniae, Haemophilus influenzae, and Moraxella catarrhalis.^[38]^ The superantigen produced by Staphylococci is a key pathogenic factor in the development of CRSwNP, and it may also contribute to GCR in patients with nasal polyps.^[39,40]^ For instance, the enterotoxin produced by Staphylococcus aureus can activate and stimulate the proliferation of CD4^+^ and CD8^+^ T cells, leading to the secretion of cytokines and inflammatory mediators,^[41]^ which triggers a cascade reaction, resulting in persistent inflammatory infiltration and edema of the nasal mucosa. In some patients with recurrent nasal polyps, there is a high proportion of GC antibody positivity, which can be induced by the enterotoxin produced by Staphylococcus aureus.^[42,43]^ This makes the body less sensitive to GC treatment, thereby reducing treatment efficacy in these patients. Some scholars have pointed out that the superantigen of Staphylococcus aureus leads to GCR by affecting the expression of GRα/GRβ. Wang et al^[44]^ confirmed that the superantigen produced by Staphylococcus aureus can lead to an increase in the expression of GRβ. Furthermore, the expression of GRβ was found to be significantly higher in recurrent nasal polyps patients who tested positive for the toxin as compared to toxin-negative CRSwNP patients. Verhaar et al^[45]^ discovered that surface superantigens of Staphylococcus aureus can trigger activation of the tyrosine kinase Lck-phospholipase C-dependent signaling pathway in T cells, causing activated T cells to exhibit GC insensitivity in vitro. The lipopolysaccharide of gram-negative bacterial products, in addition to its toxic effects, can also elicit immune responses.^[46,47]^ Fernández-Bertolín et al^[48]^ found that LPS weakens the nuclear transport capability of GRα. In addition, Cosio et al^[49]^ conducted in vitro experiments demonstrating that Haemophilus influenzae can induce GCR in chronic obstructive pulmonary diseases (COPD). Specifically, they found that this bacterium reduces HDAC activity and induces NF-κB activation, leading to the observed GC-resistant inflammatory responses. Goleva et al^[50]^ discovered that Moraxella catarrhalis can induce GC-resistant asthma through the TGF-β-related kinase 1 pathway by enhancing the expression of mitogen-activated protein kinase 1 (MAPK-1), which might also contribute to the occurrence of GCR in patients with nasal polyps.

#### 4.1.3. Bone remodeling.

Bone remodeling is the term used to describe the inflammatory changes that occur in the bone beneath the mucosa of the sinuses, including both bone destruction and new bone formation.^[51]^ In 1998, Kennedy et al^[52]^ observed ethmoid specimens under a microscope after ESS and found bone hyperplasia. They explained the phenomenon of osteitis in CRS at the histopathological level, where osteoclasts and osteogenesis are highly active, leading to bone destruction and hyperplasia. Inflammatory cell infiltration on the surface of the bone was also noted. These observations led to the use of “bone remodeling” to describe the phenomenon of osteitis in CRS. Giacchi et al^[51]^ reported that bone absorption and remodeling were observed in 18 out of 19 ethmoid bone specimens obtained from patients with CRS who underwent ESS. Richtsmeier^[53]^ identified and summarized the “top 10 reasons” for the failure of ESS in patients with CRS. Based on this analysis, bone remodeling was ranked eighth among these reasons. The study has suggested that if the inflamed bone is not removed during surgery, local inflammation may persist, and this can be considered one of the “principles” for secondary ESS.^[54]^ Matrix metalloproteinases (MMPs) are specific factors that are closely implicated in the pathogenesis of bone remodeling. Research has revealed a direct correlation between MMP9 and bone remodeling in humans, with abnormal regulation of MMP9 becoming increasingly pronounced as bone remodeling intensifies.^[55]^ However, studies have shown that even after nasal or oral GC treatment, overexpression of the MMP9 gene still persists,^[56]^ implying that MMP9 gene overexpression may not be entirely hormone-dependent. In addition, in inflammatory diseases, overexpression of MMP9 can be down-regulated by GCs, but is often accompanied by overexpression of MMP9 after the development of GCR.^[57]^ Furthermore, GC treatment may not be able to entirely halt bone remodeling.

### 4.2. Systemic factor

#### 4.2.1. Gastroesophageal reflux disease.

In recent years, various studies have revealed a correlation between CRS and gastroesophageal reflux disease (GERD). Reflux has a direct impact on the mucosa, leading to local edema and damaging the cilia clearance function of the mucosa, and the direct effect of Helicobacter pylori, as well as the possible dysfunction of autonomic nervous system function, are considered to be one of the possible factors inducing CRS and promoting its deterioration.^[58–60]^ These effects are considered to be one of the possible factors responsible for the initiation and escalation of CRS. Kim et al^[61]^ found that 17.1% of 23,489 patients with CRS also had GERD, which was significantly higher than the prevalence in the control group. A study conducted in Taiwan involved 15,807 patients who were diagnosed with GERD from January 1, 2006 to December 31, 2009.^[62]^ Additionally, 47,421 normal controls were randomly selected for comparison. After an average follow-up of 2.12 years, 964 subjects (1.52%) of CRS were identified. Among these, 406 patients (2.57%) had concurrent GERD, while 558 patients (1.18%) did not have it. The incidence of CRS among patients with GERD was found to be 2.36 times higher than that observed in normal individuals, indicating a highly significant correlation between GERD and CRS of the upper airway. Laryngopharyngeal reflux is believed to be one of the potential causes of GC-resistant CRSwNP.

#### 4.2.2. Non-steroidal anti-inflammatory drug-exacerbated respiratory disease (N-ERD).

N-ERD is a chronic eosinophilic inflammatory respiratory disorder that occurs in patients with asthma and/or CRSwNP, symptoms of which are exacerbated by non-steroidal anti-inflammatory drugs, including aspirin.^[63]^ Aspirin-exacerbated respiratory disease, a condition that includes aspirin sensitivity, asthma, and nasal polyps, typically presents with more severe asthma symptoms, diffuse and severe CRS, and a higher incidence of nasal polyp recurrence.^[63,64]^ Additionally, aspirin-exacerbated respiratory disease patients often exhibit less responsiveness to GC treatment and require surgical intervention with higher complication rates and increased postoperative recurrence rates.^[65]^ In patients with N-ERD, those with comorbid CRSwNP typically experience more severe upper respiratory symptoms, a wider range of computed tomography lesions, and more frequent nasal polyp recurrence following surgical intervention compared to non-N-ERD CRSwNP patients.^[63]^

#### 4.2.3. Immunodeficiency disease.

In the context of immunodeficient conditions, patients with CRSwNP often display a diminished response to standard drug treatments, which can ultimately lead to a significant decrease in drug sensitivity and the development of apparent drug resistance. Studies have indicated that immunodeficiency may be a potential cause of multiple relapses of CRSwNP, such as selective IgA deficiency, IgG subclass deficiency, and hypogammaglobulinemia.^[66,67]^ Possible mechanisms include T lymphocyte dysfunction, immunoglobulin deficiency or decreased function, and complement system abnormalities.^[68,69]^ Specifically, 1 study found that 23% of patients with difficult-to-treat rhinosinusitis and 13% of patients with recurrent CRS had immunoglobulin deficiencies.^[70]^ Changes in the local immune environment resulting from immunodeficiency, impaired T lymphocyte function, and immunoglobulin deficiency or dysfunction can contribute to the persistence and refractory nature of CRS.

### 4.3. Patients factors

The success or failure of treatment for CRSwNP is closely linked to patient factors. The prolonged course of the disease causes great distress for patients, which can reduce their confidence and patience for treatment. This can result in irregular follow-up visits, noncompliance with medical advice, and a lack of attention to related matters during the comprehensive perioperative treatment. These factors can contribute to the development of drug resistance and ultimately affect the patient’s recovery.

## 5. The mechanism of GCR

GCR is a common characteristic shared by airway diseases such as asthma^[44,71]^ and COPD.^[72]^ It is the primary cause of the clinical challenges associated with effectively controlling these chronic conditions. More and more researchers have begun to focus on the molecular mechanisms of GCR in these diseases and have identified certain related signaling pathways. The “integrated inflammatory response of the upper and lower airways” theory suggests that there may be shared mechanisms underlying GCR in CRSwNP and other diseases with GCR.^[73]^ Many scholars have proposed this hypothesis. Therefore, next, we will elaborate on the mechanism of GCR from the aspects of GR subtype expression, transcription level, translation level, post-transcription and post-translational processing modification, certain cells and cytokines, and gene abnormalities.

### 5.1. GR subtypes

In 1985, the cDNA sequence of the GR was first cloned in humans. The human GR gene is encoded by nuclear receptor subfamily 3 group C member 1 on chromosome 5q31–32 and consists of 9 exons. Alternative splicing of the exon 9 region can result in the production of 2 receptor subtypes, GRα and GRβ. The expression of GR subtypes is influenced by species, tissue, and cell type, thereby conferring specificity to the receptor distribution.^[74]^ Moreover, the same cell can exhibit different GR expression levels and functions under different biological states. These factors can alter GC sensitivity, ultimately affecting the response to GC treatment. The GRα subtype participates in the formation of GR homodimers and translocation into the nucleus, facilitated by zinc finger motifs that bind to GREs in target genes, and it is the main subtype mediating the effects of GC.^[75,76]^ GRβ has a distinct C-terminal sequence when compared to GRα, thereby conferring specificity to the subtype. Specifically, GRβ is unable to bind GC with specificity.^[77]^ When co-expressed with GRα in transfection studies, GRβ functions as an “endogenous inhibitor” by forming heterodimers with GRα. By competing for binding to GREs and reducing the activity of GRα, GRβ inhibits targeted transcription mediated by GRα.

GR is widely distributed in the nasal mucosa and polyp tissue.^[78]^ The existing literature suggested that the presence of an abnormal number and ratio of GR may be responsible for GCR in CRSwNP. Previous studies on GR subtypes in CRSwNP have predominantly concentrated on total GR, GRα, and/or GRβ, and the results were controversial and inconsistent.^[79,80]^ Research has demonstrated that, in certain inflammatory conditions, the expression level of GRβ may be lower than that of GRα, and this phenomenon has been postulated to be associated with GCR.^[79,81]^ Pujols and coworkers revealed an increase in protein expression of GRβ in nasal polyp tissue samples where the level of inflammatory cells exceeded 3%.^[82]^ Valera et al^[11]^ observed that there was no marked difference in GRα expression before and after nasal polyp treatment. However, they did note a decrease in GRα expression before treatment. Wang et al^[44]^ did not detect any significant variance in nucleic acid-level GRα expression amongst the CRSsNP, CRSwNP, CRSwNP relapse group, and control group. Pujols et al^[79]^ revealed that the protein expression of GR, as measured by immunohistochemistry, was diminished in nasal polyps. However, following short-term treatment with oral glucocorticoids and intranasal budesonide, the expression of GR was upregulated. Various studies have reported that compared to healthy nasal mucosa, the expression of GRα is reduced in nasal epithelial cells of nasal polyps. Additionally, the expression of GRβ mRNA remains low in all nasal specimens.^[79]^ Li et al^[31]^ discovered that the nucleic acid level of GRα expression was decreased, while that of GRβ expression was increased in nasal polyps compared to normal nasal mucosal tissue. Subsequently, CRSwNP patients were divided into GC-sensitive and resistant groups after a 1-month nasal spray GC treatment. Further studies revealed that the abnormal distribution of GR expression may be associated with different GC sensitivities of various CRS lesions. In contrast, Watanabe and Suzaki^[83]^ observed a significant increase in the expression of GRα in the infiltrated inflammatory cells of NPs in patients with asthma and found that the expression was significantly reduced after GC treatment. Choi et al^[80]^ found that the nucleic acid level of GRα expression in nasal polyps increased compared to the normal nasal mucosa and decreased after oral GC treatment. The expression level of GRβ was not significantly different before and after treatment, indicating that the expression level of GRβ is not significantly correlated with GC sensitivity. Furthermore, a recent study investigating the association between CRS and GC sensitivity found that compared to the normal control group, the expression of GRα and the GRα/GRβ ratio in peripheral blood mononuclear lymphocytes (PBML) of patients with different types of CRS decreased.^[84]^ Several studies have demonstrated that the expression of GRα mRNA significantly decreases in nasal polyps after GC treatment, while that of GRβ mRNA remains unchanged,^[83]^ suggesting that GRα may play a more significant role in nasal polyp-related inflammation. Additionally, some studies have also indicated that the enhanced expression of GRβ under disease conditions is often accompanied by GC resistance or insensitivity.^[11,79]^ Similarly, researchers have observed that the upregulation of GRβ expression in the glaucoma trabecular meshwork cells in glaucoma leads to increased resistance, while its downregulation results in increased GC sensitivity.^[85]^

The above studies highlighted the close relationship between the dysregulation of GR subtypes expression and GCR by analyzing changes in GRs, inflammatory mediators, or inflammatory cells in nasal polyp tissue before and after GC treatment. They also underscored the intricacy of the GCR mechanism in CRS, which necessitates further investigation. In addition, GCR occurs when there is an abnormality in the process of receptor binding to GREs. Studies have demonstrated that GC can operate on negative GREs located within the GR promoter, which leads to the downregulation of GRα expression.^[86]^ Consequently, GC significantly restricts signal transduction through this pathway. Research has revealed that the promoter region containing nGREs within the exon 6 region of GRα is responsible for the early-stage reduction of GRα transcription levels, and this inhibition is mainly mediated by transcriptional factors acting on nGREs.

### 5.2. GR transcription factors

The formation of CRSwNP involves numerous transcription factors, including but not limited to nuclear factor-kappa B (NF-κB), activator protein-1 (AP-1), and signal transducer and activator of transcription (STAT).^[87–90]^ These transcription factors play a key role in regulating the expression of T cells and other related inflammatory genes, thereby serving as a crucial factor in the development of GCR. Studies have demonstrated that the abnormal activation of transcription factors can contribute to the development of GCR.

#### 5.2.1. Nuclear factor-kappa B.

NF-κB is a crucial regulatory factor in inflammation that governs cell inflammatory responses and cytokine expression.^[91]^ The abnormal activation of NF-κB can prevent the binding of GR to DNA-GREs, leading to a reduction in the production of anti-inflammatory factors and ultimately causing GCR.^[92]^ Prior research has provided evidence supporting the positive correlation between the activation of NF-κB and the upregulation of downstream miR-155.^[93]^ Studies have reported that miR-155 plays a role in regulating the expression of multiple inflammatory cytokines, including TNF-α, IL-1, and IL-4.^[94,95]^ Compared to CRSsNP, the expression of miR-155 in CRSwNP is significantly elevated,^[96]^ suggesting that NF-κB is over-activated in nasal polyp tissue. The activation of the NF-κB/miR-155 signaling pathway regulates the expression pattern of cytokines, ultimately modulating the inflammatory response.^[93]^ To summarize, NF-κB activation is known to increase miR-155 expression in CRSwNP, ultimately resulting in changes in the expression of downstream inflammatory cytokines and the development of GCR.

#### 5.2.2. Signal transducer and activator of transcription 5.

STAT is a nuclear transcription factor that binds to DNA and regulates gene expression. The STAT family encompasses STAT5, which, upon phosphorylation, can form heterodimers with GR and inhibit the expression of anti-inflammatory genes. Studies have shown that when the STAT5 gene is knocked out in mouse T cells, IL-2 is unable to induce GCR.^[97]^

#### 5.2.3. Activator protein-1.

AP-1 is a transcriptional activation factor found in cells and dysregulated expression of which can result in inflammatory reactions.^[98]^ The binding of GC to the GR can result in the production of substances that inhibit the activity of AP-1. Consequently, this leads to the suppression of gene expression of inflammatory factors. Therefore, AP-1 is thought to play a crucial role in mediating the effects of drugs.

### 5.3. GR transcription subtypes

During the transcription process, every GR transcription subtype may selectively produce translation subtypes possessing distinct structures and functions, which greatly enriches the heterogeneity of the GR library and the diversity of GC signal transduction, ultimately influencing the sensitivity of target organs to GC.^[99,100]^ Taking GRα as an example, it has been found that different translation starts in exon 2 lead to the production of 8 translation subtypes, namely GRα-A, -B, -C1, -C2, -C3, -D1, -D2, and -D312. Studies suggest that the most significant difference between these GRα translation subtypes is their varying transcriptional activity.^[99,101]^ Cells that have high expression levels of GRα-C3 are more susceptible to GC-induced apoptosis, whereas cells with high expression of GRα-D3 are more likely to exhibit resistance to GC.^[99]^ Analysis indicates that the high transcriptional activity of GRα-C3 may be attributed to the direct exposure of the AF1 region, which facilitates significant recruitment of diverse co-regulators.

### 5.4. MAPK-1 signaling pathway

The MAPK family comprises a group of serine/threonine protein kinases, which primarily includes 3 subtypes: extracellular signal-regulated protein kinase (ERK), c-Jun N-terminal protein kinase (JNK), and p38 MAPK. All 3 subtypes have the function of phosphorylating GR, and their signal pathways mainly exist in cells, which can transduce external stimulus signals to the nucleus and participate in various biological processes such as cell growth, cell differentiation, and cell death.^[102,103]^

The MAPK signaling pathway plays a role in upstream signal transduction pathways and facilitates the synthesis and expression of various GR subtypes. In vitro, study has shown that macrophages from MAPK-1 knockout mice have weakened GC anti-inflammatory effects due to the activation of MAPK.^[104]^ In asthma patients with GCR, the expression of MAPK-1 in alveolar macrophages is significantly reduced after GC exposure, and this phenomenon is associated with an increase in p38 MAPK activity.^[105]^ The ERK signaling pathway, as a subclass of the MAPK pathway, is known for promoting cell proliferation and survival.^[106,107]^ In their in vitro studies on thyrotropin-releasing hormone genes, Cote-Vélez et al^[108]^ demonstrated that the ERK pathway may regulate the activity of GR by inhibiting the binding ability of GR and AP-1 on the thyroid stimulating hormone gene promoter. Li et al^[109]^ found that ERK can impede GR nuclear translocation, thereby resulting in GCR. The JNK, which is another MAPK, is activated by TNF-α and other pro-inflammatory factors, and directly phosphorylates GR at the Ser-226 position, leading to the inhibition of its binding to GREs.^[110]^ Furthermore, the study has demonstrated that the JNK pathway can elevate the nuclear export of GR by phosphorylating Ser-266, ultimately halting GR-mediated gene transcription and inhibiting its biological activity.^[111]^ The p38 MAPK pathway is extensively implicated in CRS tissue and human nasal epithelial cell lines.^[112]^ Wang et al^[113]^ discovered that p38 MAPK and JNK signaling pathways are involved in the regulation of GR subtypes expression, resulting in a reduction in the expression of GRα/GRβ. The experiments also suggest that these 2 signaling pathways are associated with the pathogenesis of GC-resistant CRS. Matthews et al^[75]^ observed that the number of IL-2 and IL-4 mRNA-expressing cells in the bronchoalveolar lavage fluid from GCR patients with asthma was higher than that in GC-sensitive patients. IL-2 and IL-4 were found to inhibit the binding of the GRα to DNA, leading to GR phosphorylation and subsequent GCR. The phosphorylation of GR in this process is mainly mediated by the p38 MAPK pathway. Irusen et al^[114]^ similarly suggested that the p38 MAPK pathway could be linked to changes in GR activity through in vivo and in vitro studies in asthma, respectively, confirming that p38 MAPK inhibitors can restore GC sensitivity in GC-resistant asthmatics.

### 5.5. Histone modification

Histone modification is a covalent post-translational modification of proteins that plays a crucial role in the gene transcription process. It involves various internal and external stimuli that lead to post-translational modifications of GR, further fine-tuning the expression and functional changes of GR. The main types of histone modification include phosphorylation, acetylation, ubiquitination, and SUMOylation of proteins.^[115,116]^ Currently, the most extensively studied covalent modifications of GR are phosphorylation and acetylation.

#### 5.5.1. Phosphorylation of GR.

GR can be phosphorylated by various kinases, resulting in changes to its binding affinity, stability, ability for nuclear translocation, and DNA binding ability, rendering it unable to exert biological effects, ultimately leading to GCR. In human, rat, and mouse GRs, there are at least 7 serine residues (Ser-113, Ser-134, Ser-141, Ser-203, Ser-211, Ser-226, and Ser-404) that can be phosphorylated.^[81,116]^ Additionally, other sites such as Ser-45 and Ser-267 can also undergo phosphorylation. When unbound to the GC, GR exists in a low phosphorylation state, which switches to a high phosphorylation state when it binds to GC. Phosphorylation of GRα can have a gene-specific effect on its transcriptional activity. Phosphorylation of the ligand-dependent Ser-211 site can increase the transcriptional activity of GRα, whereas phosphorylation of the Ser-226 site can reduce its transcriptional activity.^[117,118]^ Phosphorylation of Ser-203, Ser-226, or Ser-404 sites can impair GR nuclear transport, consequently causing a reduction in its transcriptional activity.^[81]^

#### 5.5.2. Acetylation of GR.

The process of histone acetylation and deacetylation is closely linked to gene activation. Deacetylation of nucleosomal core histones alters the chromatin structure, exposing DNA transcription binding sites, and thus facilitating transcription. Acetylation, conversely, inhibits transcription. Histone deacetylases (HDACs) are a type of acetylase that are primarily distributed in the nucleus of cells. HDACs can regulate the NF-κB and TLR4-MyD88 signaling pathways by promoting histone deacetylation, which modifies the biological activity of many transcription factors, receptors, and histones, ultimately exerting anti-inflammatory effects and participation in the body’s inflammatory response.^[119]^

The expression level and activity of HDAC are closely associated with its pro-inflammatory and anti-inflammatory effects. There are currently 3 main types of HDACs, comprising 11 subtypes, each with differing activities.^[120]^ HDAC levels are regulated by histone acetyltransferases and HDACs.^[121]^ Among the multiple subtypes of HDAC, HDAC1 has been extensively studied. A decrease in HDAC1 expression disrupts the dynamic balance between histone acetyltransferases and HDACs, ultimately resulting in histone deacetylation, an increase in inflammation-related gene transcription, the synthesis of inflammatory proteins, and a reduction in GC anti-inflammatory effects. In addition, decreased HDAC1 expression can cause acetylation of deacetylated NF-κB, leading to increased NF-κB activity.^[122]^ This interferes with the ability of activated GC to bind to GREs, thereby diminishing GC’s anti-inflammatory effects and resulting in GCR. HDAC2 and HDAC3 have been shown to possess anti-inflammatory properties by repressing the expression of pro-inflammatory genes. Specifically, HDAC2 promotes histone deacetylation and activates GR-induced transcription, which is a critical mechanism of GC action. In some patients with poor GC response, reduced HDAC2 expression and activity have been found. In a mouse model of asthma, persistent inflammatory reactions and oxidative stress led to a decrease in HDAC2 expression, which resulted in GCR.^[123]^ Oxidative stress limits GC recruitment to GR gene sites by inhibiting the expression and activity of HDAC2, which can decrease the effectiveness of GCs.^[124]^ Similarly, GRβ can reduce the sensitivity of GCs by inhibiting the transcription initiation of GREs and decreasing HDAC2 function.^[125]^ Ito et al^[126]^ also reported similar findings, suggesting that HDAC2 suppresses the activity of NF-κB and thus plays a pivotal role in the development of GCR. Moreover, the activation of neutrophils and oxidative stress triggered by cigarette smoke are major factors contributing to GCR through HDAC modulation,^[72]^ suggesting that smoking may also be a significant cause of GCR in patients with nasal polyps.

### 5.6. Mucin (MUC) subtypes

MUC is a significant component of the mucus layer lining the airway epithelium, and it plays a crucial role in protecting the sinonasal mucosa by facilitating the mucociliary clearance mechanism, which helps eliminate pathogens and irritants from the nasal cavity.^[127,128]^ Also, recent evidence indicates that MUC has additional functions in host defense against respiratory infections and inflammation, as well as in morphogenesis, repair, and treatment responses.^[129,130]^ As per the literature, MUC subtypes in nasal polyp tissue can interact with GR, thereby affecting the biological effects of GC.^[131]^ GR protein forms a complex with MUC, localized in the nucleus, to exert dexamethasone’s anti-inflammatory effects. Milara et al^[130]^ found that in GC-resistant CRSwNP patients, the expression of MUC1 was reduced, and there was a decrease in the formation of the GRα complex and an increase in serine residue phosphorylation at the GRα 226 site, which ultimately resulted in reduced nuclear translocation of GR and anti-inflammatory effects, leading to GCR. Subsequent studies by Milara et al^[32]^ found that MUC4 is also capable of binding to the GRα. Interestingly, the MUC4-GRα complex was significantly increased in patients with GCR in nasal polyps, indicating that overexpression of MUC4 may contribute to GCR development in these patients.

### 5.7. Neutrophils

An increasing body of evidence indicates that 65% to 90% of CRSwNP patients in Europe and the United States present with eosinophilic inflammation,^[132]^ while patients in East Asian countries mainly exhibit neutrophil inflammation.^[4]^ Neutrophil migration and activation in CRSwNP have been linked to Staphylococcus aureus colonization.^[133]^ Activated neutrophils play a vital role in phagocytizing Staphylococcus aureus and initiating antimicrobial cascades and oxidative bursts, which contribute to airway hyperresponsiveness.^[134]^ Neutrophilic inflammation is a common feature in various GCR diseases, often characterized by an increase in neutrophil counts.^[135]^ Increased peripheral blood neutrophil counts have been observed in GC-resistant asthmatics, and patients with high-neutrophil nasal polyps have been shown to exhibit reduced GC responsiveness.^[135,136]^ In CRSwNP patients, treatment with GCs led to a significant decrease in levels of eosinophil cationic protein, IL-4, IL-5, and other related indicators in nasal secretions. However, no significant changes were observed in neutrophil counts or their related cytokine levels.^[135]^ GCs are not effective in treating neutrophilic inflammation in asthma and may even exacerbate its inflammatory severity.^[137]^ Likewise, Liao’s clustering study reported that neutrophilic inflammation exhibited a poor response to corticosteroid treatment.^[138]^ Furthermore, the study identified a high distribution of GRβ on neutrophils, and an increase in GRβ numbers may significantly reduce the anti-inflammatory effects mediated by GRα.^[139]^ This may contribute to GCR in nasal polyps characterized by neutrophil infiltration. Overall, the aforementioned findings suggest that GCs can effectively suppress the function of eosinophils, but they may be insufficient in inhibiting the inflammatory reaction caused by neutrophils.

### 5.8. T cells and cytokines

The immunological mechanism is a crucial component of the pathogenesis of CRSwNP, with GCs acting by modulating CD4^+^ T cells and inflammatory factors. In recent years, there has been a growing interest among scholars in investigating the involvement of Th17 cells and IL-17 in GCRs. Th17 cells are primarily responsible for producing a range of cytokines, including IL-17A, IL-17F, and IL-21, and participate in the immune response of resisting extracellular pathogens.^[140,141]^ Studies have found that local high expression of IL-17 in CRSwNP tissues is positively correlated with local neutrophil infiltration.^[132,142]^ McKinley et al^[143]^ found that GCs could inhibit the secretion of IL-5 and IL-13 by Th2 cells, thereby curtailing the inflammatory response, while they were unable to suppress the production of cytokines (such as IL-17) or airway inflammation caused by Th17 cells. In vitro, study has indicated that GCs cannot impact the production of IL-17A and IL-22 by Th17 cells.^[143]^ Murcia et al^[144]^ conducted experiments in which they stimulated neutrophils with IL-17 and found that it had a direct activating effect, causing the production of IL-8. They also found that IL-8 produced in this way was not inhibited by GC. In addition, IL-17 was observed to stimulate endothelial cells, leading to an increase in neutrophil adhesion to neutrophils. Taken together, these findings suggest that IL-17 can effectively trigger inflammation in endothelial cells, resulting in the infiltration of neutrophils and resulting in GCR.^[145]^ Blocking the effect of IL-17 on endothelial cells and preventing neutrophil recruitment are beneficial in the treatment of neutrophilic inflammatory diseases.^[120]^ Based on the prior studies, various cytokine stimuli can also alter the expression and binding affinity of GRα and GRβ, which can contribute to the development of GCR. Lavender^[146]^ proposes that IL-17 can induce a downregulation of GRα expression and an upregulation of GRβ expression within epithelial cells, which may lead to GC insensitivity. Wang et al^[113]^ validated in vitro that IL-1β can decrease the ratio of GRα/GRβ in nasal polyp epithelium via the induction of p38 MAPK, which can contribute to GCR in nasal polyps. And, blocking of the p38 MAPK and JNK pathways can increase the ratio of GRα/GRβ and enhance the affinity of GR.

### 5.9. 11β-hydroxysteroid dehydrogenase

Hormone activity enzymes have an impact on the concentration and biological utilization of active hormones within cells or tissues, which consequently affect their anti-inflammatory effects. The hormone activity enzyme 11β-hydroxysteroid dehydrogenase (11β-HSD) includes 2 subtypes, 11β-HSD1 and 11β-HSD2, both of which are primarily expressed in the endoplasmic reticulum of cells. These enzymes play a crucial role in regulating the activation state of hormones in tissues. Existing literature suggests that an unbalanced expression of 11β-HSD1 and 11β-HSD2 can result in reduced levels of active hormones within cells, which can ultimately affect the treatment of chronic inflammatory diseases.^[147,148]^ 11β-HSD1 is involved in the conversion of inactive hormones to active hormones, thus leading to an increase in endogenous GC biological activity within the nasal mucosa. Augmenting the activity of 11β-HSD1 may represent a promising therapeutic strategy for the treatment of GC-resistant patients.^[89]^ Conversely, 11β-HSD2 exerts an opposite effect.^[149]^ As per Jun et al^[148]^ reported that both 11β-HSD1 and 11β-HSD2 enzymes are expressed in the nasal mucosa, with the former being significantly increased and the latter decreased in the sinus mucosa of CRSwNP patients. The expression of these 2 enzymes is modulated by inflammatory cells present locally, whereas nasal mucosal epithelial cells are also influenced by the hypothalamic-pituitary-adrenal axis in regulating the production of local endogenous hormones. In addition, Prodanovic et al^[150]^ proposed that endogenous hormones produced locally can potentially curtail the efficacy of synthetic hormones in airway epithelial cells. The cellular 11β-HSD-mediated regulation of local endogenous hormone levels may contribute to the hormone insensitivity observed in CRSwNP, among other factors. More recently, Jiang et al^[151]^ found a noteworthy association between the expression of 11β-HSD and the response of nasal polyps to hormone therapy, and the expression ratio of 11β-HSD was identified as a crucial biomarker for predicting GC sensitivity in nasal polyps. Taken together, these findings suggest that an imbalance of 11β-HSD expression can significantly limit the efficacy of GC treatment in nasal polyps.

### 5.10. Molecular chaperone

Upon binding to the GR, the transcriptional response of GCs requires a mature and intact GR complex. Disruptions in the molecular chaperones that make up the GR complex can potentially alter the sensitivity of GCs. HSP90 is an important molecular chaperone that ensures the normal function of GR. Lauten et al^[152]^ found that leucocytes of patients with acute lymphoblastic leukemia exhibit abnormal levels of HSP90 and HSP70. Studies have demonstrated that alterations in the expression levels of HSP90 may contribute to GCR observed in asthma, multiple sclerosis, and congenital nephrotic syndrome. Broennegaard and Carlstedt-Duke^[153]^ found that increased expression of HSP90β mRNA can decrease the receptor’s affinity to GC. Moreover, Denny et al^[154]^ discovered that FKBP52 acts as a positive regulator of GC, while FKBP51 acts as a negative regulator of GC activity by reducing the receptor’s affinity. The expression levels of FKBP51 and FKBP52 are associated with changes in GC sensitivity. Recent research has revealed that in obese children with asthma, FKBP51 expression in CD4^+^ T-Lymphocytes induced by dexamethasone is associated with worsening asthma control, which may underline the occurrence of GCR.^[155]^

### 5.11. GR gene polymorphism and gene mutations

Currently, it is known that variations in GR genes, including GLCCI1(rs37973), Tth111l (rs10052957), BclI (rs41423247), ER22/23EK (rs6189/rs6190), N363S (rs6195), and 9β (rs6198), may be associated with sensitivity pattern and different patterns of GCs response.^[156–158]^ A preliminary study found a significant association between gene polymorphism and GCR in patients with primary nephrotic syndrome.^[159]^ Mutations in genes can disrupt the process of signal transduction between GCs and target cells, resulting in reduced potency and sensitivity of GCs. In vitro studies and investigations on healthy individuals have demonstrated that mutations in the nuclear receptor subfamily 3 group C member 1 gene can regulate individual sensitivity to GCs.^[160]^ Charmandari et al^[161]^ identified an exon 9 mutation in the GRα gene in a patient who developed GCR, where leucine was replaced by proline at amino acid position 773 in the GRα ligand-binding domain. This mutation reduced the GR’s affinity to GC and, as a result, affected the GCs’ effectiveness. Interestingly, Hawkins et al^[162]^ reported that changes in GC sensitivity in asthma patients were not correlated with polymorphisms in the FKBP5 gene. On the other hand, gene variations encoding HSP70/HSP90 assembly proteins (STIP1) were found to increase GC sensitivity in asthma patients.

### 5.12. MiRNAs

After GR transcription, regulation primarily takes place in the 3’-untranslated region of GR nucleic acid, which fine-tunes the post-transcriptional regulation of GR by affecting the stability of mature receptor mRNA levels and GR protein expression. It has been reported that some miRNAs play an important role in post-transcriptional regulation. MiRNAs are a class of non-coding RNAs that play a crucial regulatory role in gene expression by inhibiting mRNA translation and triggering mRNA degradation. Abnormalities in certain miRNAs might be linked to the development of GCR. Recent research suggests that elevated levels of miR-124, miR-130b, and miR-142-3p in humans can result in decreased expression levels of GRα nucleic acid and protein.^[163–165]^ Kotani et al^[166]^ reported a genetic mutation, in a patient with MLL-AF4 ALL, which caused a reduction in miRNA128b expression levels, resulting in GCR.

## 6. Future strategies of GCR

To date, the pathogenic factors of GC-resistant CRSwNP are multifactorial and the underlying pathological and physiological mechanisms remain incompletely clear. Consequently, treatment options for these patients are currently limited, presenting a significant clinical challenge. As of now, there is no standardized treatment plan for GCR patients with CRSwNP. The current strategy for management involves implementing systematic and individualized comprehensive treatment, primarily based on FESS. Additionally, the treatment may incorporate alternative anti-inflammatory drugs and molecular pathways to reverse GCR and thereby relieve clinical symptoms experienced by patients with GCR.

Apart from surgical intervention, the emergence and development of targeted therapeutic drugs have introduced a new avenue for treating CRSwNP. This has led to the concept of “pharmacological polyp removal,” which is becoming increasingly plausible. Clinical studies have demonstrated that biologics can significantly reduce the size of nasal polyps and alleviate nasal congestion.^[167,168]^ Moreover, they have been found to improve olfactory function and thereby enhance the quality of life of GC-resistant CRSwNP patients. Also, the use of biologics may decrease the proportion of oral glucocorticoid use and reoperation rate, providing a new treatment for patients with GCR.^[169]^ Currently, several types of monoclonal antibodies can be considered for use in GC-resistant CRSwNP, including anti-IL-5 monoclonal antibodies (such as Mepolizumab), anti-IL-4/13 (such as Dupilumab), and anti-IgE monoclonal antibodies (such as Omalizumab).^[170–172]^ What’s more, there are also biologics such as monoclonal antibodies against thymic stromal lymphopoietin, IL-25, IL-33, and eosinophilic chemotactic factors that require further clinical research. While research suggests that immunomodulatory agents are promising treatments for patients with CRSwNP, challenges remain in the clinical application. Specifically, rigorous evaluations are necessary due to 2 prominent issues. Firstly, the efficacy of such treatment is variable, with only 50% to 70% of patients showing positive responses to biologics. Secondly, the high cost of immunotherapy should be considered, as it may pose an economic burden to patients. Recent studies suggest that the annual cost of biologics treatment in the United States ranges from $31,000 to $40,000,^[173]^ whereas the average cost per endoscopic sinus surgery ranges from $8000 to $11,000.^[174]^ In terms of cost-effectiveness, surgical treatment appears to have a more favorable profile than biologics treatment.^[175]^ Additionally, the latter is more time-consuming and expensive, making it more challenging to be widely applied.^[176]^

Recent studies have shown that vitamin D (VD) plays an important role in calcium and phosphorus metabolism and also immune regulation.^[177]^ VD has been found to modulate the inflammatory cytokine profile released by T cells, which can inhibit Th1 and Th17 cell inflammation, induce Treg cells to secrete IL-10, and enhance the Th2 immune response.^[178–180]^ These effects contribute to an increase in the anti-inflammatory effect of GCs. Research has found that VD and GCs have individual effects on airway smooth muscle cells, leading to a decrease in the proportion of inflammatory chemokines by 38% and 20%, respectively. When used in combination, the reduction reached 60%,^[181]^ indicating that VD can enhance patients’ sensitivity to GC. Fraczek et al^[182]^ concluded that VD might improve GC sensitivity in the treatment of CRSwNP to a certain extent by detecting the level of chemokine reduced upon activation, normal T-cell expressed and secreted. In addition, Wang et al^[183]^ proved that VD can significantly inhibit the production of eotaxin and reduced upon activation, normal T-cell expressed and secreted stimulated by IL-1β in nasal polyp tissues. Another study by Wang et al^[184]^ showed that VD can effectively inhibit the production of MMP-2 and MMP-9 in nasal polyp tissues stimulated by TNF-α. These 2 proteases play a significant role in tissue remodeling, which is crucial for the treatment of GC-resistant CRSwNP. Studies have also reported that the combination of VD and GC can effectively reduce the activation of the NF-κB pathway and alleviate inflammatory reactions induced by LPS.^[185]^ Shymanskyi et al^[186]^ demonstrated that VD supplementation can improve GR abnormalities resulting from chronic GC administration, suggesting that VD may partially reverse GCR. However, real-world evidence is necessary to determine the optimal serum VD concentration, the appropriate dosage of VD supplementation to enhance the anti-inflammatory effects of GCs, the optimal synergy between the two, as well as the treatment duration and potential adverse reactions in the treatment of CRSwNP. What’s more, recent studies have indicated that inhibitors of P-glycoprotein exhibit comparable therapeutic effects to oral GCs and biologics in the treatment of nasal polyps^[187–189]^ indicating that P-glycoprotein inhibitory function might serve as alternative drugs to GCs for treating GC-resistant patients with nasal polyps. Macrolide antibiotics have been proven effective in treating CRS patients with GCR.^[190,191]^ The study has demonstrated that theophylline can effectively restore HDAC2 activity in macrophages of patients with COPD to normal levels, thus reversing GCR.^[192]^ After being exposed to cigarette smoke, mice exhibiting GC-resistant inflammatory reactions have shown a reversal of their symptoms upon administration of theophylline. However, further research is required to determine whether theophylline can be effectively used in the treatment of GC-resistant nasal polyps. In the latest study, Jiang et al^[193]^ discovered that NF-κB inhibitors and p38 MAPK inhibitors can effectively reduce the expression of TNF-α and IL-6 mRNA in nasal epithelial cells of patients with CRSwNP. Additionally, these inhibitors increase the level of GRα, indicating their potential utility in treating neutrophilic nasal polyps. There is compelling evidence supporting obesity as an independent risk factor for CRS.^[194]^ The inflammatory state associated with excess adipose tissue appears to create a favorable environment for the development of CRS. Furthermore, it has been observed that overweight and obese individuals may experience neutrophil infiltration in the body, which can contribute to insensitivity to GC treatment.^[195,196]^ Zhang et al^[197]^ first demonstrated that overweight and obesity are independent risk factors for GC insensitivity in patients with CRSwNP, suggesting that weight management may serve as a potential strategy for improving treatment outcomes in GC-resistant individuals who are overweight or obese.

## 7. Conclusion

This article primarily focuses on reviewing the potential pathogenic factors associated with GC-resistant CRSwNP patients, as well as the underlying mechanisms and treatment strategies for GCR. The treatment of CRSwNP patients with GCR is currently a prominent area of research. It is evident that drug treatments often yield poor results in GC-insensitive patients, and postoperative recurrence is frequently observed, which causes an immense burden associated with medical costs and healthcare. Indeed, the intricate signal transduction mechanism of GCR in CRSwNP, as well as the complex interactions between various signaling pathways, merit further investigation. It is important to delve deeper into these aspects to establish a comprehensive and unified understanding. Therefore, on the 1 hand, by conducting an in-depth study of the pathogenic factors and mechanism of GCR in CRSwNP, we can discover new insights into the pathological mechanism of CRSwNP with GCR. These findings can provide an experimental basis and valuable scientific clues for enhancing the efficacy of GCR treatment and exploring more precise treatment approaches for CRSwNP as a whole. This serves as the fundamental basis for the prevention and treatment of GC-insensitive CRSwNP. While on the other hand, CRSwNP is a persistent condition that necessitates long-term self-management and collaborative efforts between healthcare professionals and patients. Enhancing patients’ knowledge and comprehension of the disease, embracing standardized diagnosis and treatment protocols, improving treatment adherence, and collectively advocating clinical standards in diagnosis and treatment will contribute to the overall improvement of human health.

## Author contributions

**Writing – original draft:** Langlang Chen, Xin Fan, Lina Yang, Lu Han, Ningbo Wang, Ka Bian.

**Writing – review & editing:** Langlang Chen, Ka Bian.
